# Retraction Note: Identification and assessment of differentially expressed necroptosis long non-coding RNAs associated with periodontitis in human

**DOI:** 10.1186/s12903-024-05157-x

**Published:** 2024-11-08

**Authors:** Jiangfeng He, Zhanglong Zheng, Sijin Li, Chongshan Liao, Yongming Li

**Affiliations:** 1 Department of Orthodontics, Stomatological Hospital and Dental School of Tongji University, Shanghai Engineering Research Center of Tooth Restoration and Regeneration, Shanghai, 200072 China; 2 Department of Maxillofacial Surgery, Stomatological Hospital and Dental School of Tongji University, Shanghai Engineering Research Center of Tooth Restoration and Regeneration, Shanghai, 200072 China

**Retraction Note: BMC Oral Health (2023) 23:632**

10.1186/s12903-023-03308-0

The Editor has retracted this article after concerns were raised about the data reported. The two datasets used in this analysis, GSE10334 and GSE16134, were each generated from the same archive of healthy and diseased gingival tissues, and as such there is a significant sampling overlap between the two. Furthermore, both datasets contain multiple samples per patient. The authors performed RT-qPCR experiments on a selection of samples to validate the findings of their bioinformatic analysis, but these experiments were conducted on LPS-treated tissue rather than on disease-state tissue, and as such do not fully mitigate the underlying limitations with these datasets. The Editor no longer has confidence in the reliability of this article’s findings or conclusions.

The authors did not respond to correspondence from the Publisher regarding this retraction.

